# Risk factors for criminal recidivism – a prospective follow-up study in prisoners with substance abuse

**DOI:** 10.1186/1471-244X-12-111

**Published:** 2012-08-15

**Authors:** Anders Håkansson, Mats Berglund

**Affiliations:** 1 Division of Psychiatry, Dept of Clinical Sciences Lund, Lund University, Lund University Hospital, Kioskgatan 17, S- 221 00, Lund, Sweden

**Keywords:** Criminal justice, Criminal recidivism, Prison, Substance use disorders, Criminal behaviour

## Abstract

**Background:**

Substance use in general has been shown to predict criminal recidivism. The present study aimed to examine potential predictors of criminal recidivism, including substance-specific substance use patterns, in prisoners with substance use.

**Methods:**

A cohort of prisoners with substance use problems (N = 4,152) were assessed with the Addiction Severity Index (ASI) in the Swedish criminal justice system. Clients were followed for an average of 2.7 years. Criminal recidivism was defined as any return to the criminal justice system.

**Results:**

During follow-up, 69 percent (n = 2,862) returned to the criminal justice system. Recidivism was associated with amphetamine and heroin use, with an additive risk for injectors, and with polysubstance use. Also, recidivism was negatively associated with alcohol, other opioids than heroin/methadone and with hallucinogenic drugs, and positively associated with previous psychiatric in-patient treatment, violent behaviour, and with a shorter index sentence. Associations remained when controlling for type of crime.

**Conclusions:**

Even when controlling for type and severity of crime, and for psychiatric problems, risk of criminal relapse was increased by substance use variables, including amphetamine, heroin and polysubstance use, and an additional risk was shown for injection drug users. These findings have implications for the need for substance abuse treatment after release from prison.

## Background

Substance abuse and criminal behaviour are closely related, and a large proportion of substance users commit crimes [1,2]. While some part of criminal behaviour is likely to occur in order to finance drug use [3], substance use is also clearly associated with violent crime [4].

Also, criminal behaviour is generally more common among mentally ill [5], and a very large proportion of criminal justice clients suffer from alcohol or drug dependence or other mental disorders, including high rates of personality disorders [6,7].

There is a growing amount of literature addressing risk factors for criminal recidivism [8-13]. A history of substance use repeatedly has been reported as a predictor of committing new offenses [8,10], and in a large meta-analysis [8], other predictors of general criminal recidivism were previous criminal history and previous violent behaviour, although a violent index crime was rather associated with a lower risk of recidivism. Other predictors were, among others, psychiatric hospital admissions, poor living conditions, male gender, younger age, and antisocial personality disorder. In this meta-analysis, being a mentally disordered offender was actually associated with a lowered risk of committing a new offense [8], although other research has demonstrated an increased risk of multiple incarcerations in clients with major psychiatric disorders, including an increased risk of repeat incarcerations in clients with bipolar disorder, psychotic disorders, or depression [9].

While the role of substance use in the prediction of criminal recidivism has been quite clearly demonstrated [8,10], there are more limited data describing how this is related to different types of substance use patterns. Bonta and colleagues [8] showed that the increased risk of recidivism was more pronounced for drugs than for alcohol, and in treatment participants in Australia, it was reported that criminal recidivism was more common in users of other drugs than cannabis, with a higher risk reported for heroin users than for methamphetamine users [11].

The present prospective follow-up aimed to identify predictors of general criminal recidivism among substance users in prison, with an emphasis on potential risk factors related to specific substance use pattern, while controlling for psychiatric problem variables and severity and type of criminality.

## Methods

### Data material

Since 2001, the Swedish Prison and Probation Service examines, to an increasing extent, clients with known or suspected substance use problems with the Addiction Severity Index [14]. The Addiction Severity Index (ASI) [15-17] is a well-documented instrument for the assessment of substance use and related problems. It is well established as a tool in addiction research, and has acceptable validity [15-19]. Annually, around 10,000 clients enter the Swedish prison system, and around 66 percent of prisoners are estimated to suffer from a substance abuse problem. The present analysis is based on ASI assessments carried out in prisoners from 2001 until the first half of 2006. During these first years of ASI use in Swedish criminal justice clients, the Swedish criminal justice system has gradually increased the use of this interview assessment, to an increasing extent and on an increasing number of correctional units nationally. Where the ASI assessment had been implemented, the assessment was made for clients with a known or suspected substance use problem, aiming to improve individual problem assessment and adequate allocation to treatment, and in order to facilitate research and quality improvement on a national level. During these years, 5,122 prisoners were assessed with the ASI, and their interview data were blinded and stored in the present database.

ASI interviews from this database have been studied in several previous analyses assessing substance use and related problems among criminal justice clients [20-22]. The last decade has seen an increasing focus on substance use in the Swedish criminal justice system, with an emphasis on illicit drug use, which is reflected in the present data material; compared to the criminal justice population as a whole, illicit drug users are over-represented (compared to problematic alcohol users), as well as women, and prisoners compared to clients serving non-custodial sentences.

In the criminal justice system, the ASI version used was the ASI-X (X for ‘extended’, [23]), which is a further development of the European standard version EuropASI [24], but similar to older standard versions of the instrument. A smaller number of younger clients were instead assessed with the similar instrument intended for adolescents, ADAD (Adolescent Drug Abuse Diagnosis, [25]).

The present study is based on baseline data from the database of ASI assessments collected between 2001 and August 2006, which contains 7,493 interviews with 7,085 clients (some clients have been re-assessed upon re-incarceration). For clients assessed more than once, in the present study the first ASI examination was used. Seventy-two percent of clients were assessed in prison (n = 5,122), while the remaining clients were interviewed while on probation (17 percent), on remand (5 percent), or in other types of correctional facilities, such as treatment institutions. A post hoc analysis indicated that around 5 to 6 percent of clients addressed for an ASI interview had refused. The time elapsed until the interview, from entry to the criminal justice unit where the interview took place, was 65 days on average (median 28 days), and 90 percent of clients were interviewed within the first year and 97 percent within two years.

In the criminal justice registry used here for data on the type of crime, the termination of the index sentence and for recidivism, these data are systematic only for prison clients, and therefore, only clients assessed in prison (N = 5,122) were included in the present analyses. In order to assess only subjects with a confirmed substance use problems, clients were included only if their baseline interview provided information about a primary drug (defined as the ‘dominating problem’ in the ASI, n = 4,275). Among them, clients were excluded if they were still in prison when relapse data were retrieved (n = 41), if the date of release from prison was unavailable (n = 48), if they died during the sentence (n = 18), or if it was reported that they interrupted or refused the ASI interview, if they were considered unable to respond adequately, if their answers were severely distorted by the client’s misrepresentation or inability to understand questions (n = 16), leaving 4,152 clients who were included in further analysis.

### Design

The present project combines data from the present ASI database with data from the Swedish criminal justice registry, in order to examine the association between baseline data from the ASI interview and prospective criminal recidivism defined as a return to the criminal justice system. The Swedish criminal justice registry collects data on sentences served in prison, on remand, or different types of non-custodial sentences, and the registry previously has been used for the prospective study of criminal acts [26]. The registry also provides data on the crimes included in the verdict.

In the present analysis, criminal recidivism was defined as any return to the criminal justice system, as indicated by the criminal justice registry. Time at risk for returning was measured from the termination of the index sentence until return or until data were censored at the end of the study (November 2010), or until time of death. ASI items describing clients’ substance use patterns were included as potential predictors of recidivism. Here, we included variables describing a lifetime history of substance use as defined in the ASI; regular use for at least one year (six months or more is approximated to one year), where regular use is defined as three or more days of use per week. For each substance included, a dichotomous variable was constructed, defining whether regular history of using the substance was reported or not.

History of injection drug use, reported in the ASI with the corresponding definition, was also assessed. As amphetamine and heroin are, by far, the substances most commonly injected by Swedish drug users and in the present dataset [21], we chose to analyze these variables together, constructing a categorical variable describing amphetamine and/or heroin use with or without the reporting of injection drug use. The absence of both amphetamine, heroin and injection drug use was used as the category of reference (Table 1). Other substances assessed for lifetime use were binge drinking, other opioids (other than heroin or methadone), sedatives, cannabis, cocaine, and hallucinogenic drugs. Binge drinking is defined in the ASI as the drinking of at least five (men) or four (women) standard drinks on the same occasion, and a history of regular binge drinking reported here is defined as this drinking pattern occurring on at least three days a week. Based on previous literature indicating an association between criminal recidivism and variables such as gender, living conditions, psychiatric problems and index criminality [8], ASI items describing these factors were included as potential predictors of criminal recidivism. This includes gender and homelessness last 30 days before incarceration, as well as a lifetime history of the different psychiatric problems (either during the last 30 days before incarceration or before during lifetime) included in the ASI interview; hospitalization, depression, anxiety, suicide attempts, difficulty controlling violent behaviour, difficulty understanding, remembering or concentrating (cognitive problems), and hallucinations. Due to its close connection to suicide attempts, suicidal ideation was excluded from regression analysis. Given the possible link between baseline criminality and future criminal recidivism, we included dichotomous information from the criminal justice registry about the principal crime in the index verdict for three major types of crime; violent crime, property crime (stealing, shoplifting, robbery), and drug crime. Also, as a theoretical proxy variable of severity of the index crime, we included the duration of the index sentence in the model, defined as the number of days, coded in intervals of 100 days; 0–99 days, 100–199 days, 200–299 days, etc. Also, in order to assess the potential role of polysubstance use, based on previous data from this database indicating a more severe clinical picture in clients reporting a higher number of substances [22], we included the number of substance types used during the last 30 days before incarceration. This variable was calculated as the number of substance groups (0 to 6) used among alcohol, opiates/opioids, sedatives, cannabis, central stimulants, or hallucinogenic drugs. In addition, the survival analysis included age at baseline.

**Table 1:** Characteristics of clients with and without return to the criminal justice system during follow-up

	**Clients relapsing, n (%)**	**Clients not relapsing, n (%)**
	**n = 2,862**	**n = 1,290**
**Demographic data**		
Mean age (yrs)	33.3	33.8
Male gender	2,607 (91)	1,133 (88)
Homelessness last 30 days	742 (26)	197 (15)
**Lifetime history of psychiatric problems**		
Hospitalization	493 (17)	169 (13)
Suicide attempt	687 (24)	269 (21)
Depression	1,472 (51)	668 (52)
Anxiety	1,583 (55)	704 (55)
Cognitive problems	1,646 (58)	686 (53)
Hallucinations	413 (14)	162 (13)
Difficulty controlling violent behaviour	1,347 (47)	500 (39)
**Lifetime history of substance use (>6 months)**		
Amphetamine, heroin and/or injection drug use		
- none	604 (21)	499 (39)
- injecting, no heroin/amphetamine	77 (3)	34 (3)
- heroin use, no injecting	69 (2)	39 (3)
- heroin use, injecting	143 (5)	44 (3)
- amphetamine use, no injecting	430 (15)	249 (19)
- amphetamine, injecting	1,050 (37)	297 (23)
- heroin and amphetamine, no injecting	50 (2)	19 (1)
- heroin and amphetamine, injecting	439 (15)	109 (8)
Binge drinking	1,307 (46)	590 (46)
Other opioids than heroin/methadone	438 (15)	177 (14)
Sedatives	1,070 (37)	405 (31)
Cocaine	455 (16)	225 (17)
Cannabis	1,815 (63)	762 (59)
Hallucinogenic drugs	365 (13)	167 (13)
**Number of substance types used last 30 days (0–6)**		
0	280 (10)	224 (17)
1	965 (34)	485 (38)
2	825 (29)	287 (22)
3	499 (17)	197 (15)
4	227 (8)	69 (5)
5	60 (2)	22 (2)
6	6 (0)	6 (0)
Mean number of substance types	1.87	1.61
**Duration of index sentence (mean number of days)**		
−99	427 (15)	104 (8)
100-199	838 (29)	237 (18)
200-299	539 (19)	187 (14)
300-399	310 (11)	153 (12)
400-499	195 (7)	95 (7)
500-599	153 (5)	84 (7)
600-699	96 (3)	72 (6)
700-799	88 (3)	83 (6)
800-899	55 (2)	47 (4)
900-999	48 (2)	43 (3)
1000-	113 (4)	185 (14)
Mean duration of index sentence (days)	326	541
**Main crime in index verdict**		
Violent crime	395 (14)	283 (22)
Property crime	976 (34)	256 (20)
Drug crime	814 (28)	501 (39)

Deaths were available from the National Death Registry for clients deceased until December 31, 2008. Additional deaths after this date (and for four individuals whose deaths were not registered in the National Death Registry), were retrieved from the criminal justice registry. The study was approved by the Ethics Committee of Lund University.

### Statistical analysis

After calculating the prevalence of each variable for clients with or without recidivism, respectively (Table 1), all variables included were entered into a Cox regression survival analysis (Table 2). Here, all variables except the type of crime were entered simultaneously, in a first model. In the second model, the three main types of crime were included, aiming to study statistical associations when controlling for type of criminal behaviour. Results are reported as hazard ratios with a 95 percent confidence interval.

**Table 2:** Variables associated with return to the criminal justice system. Hierarchical Cox regression analysis

	**Model 1**	**Model 2**
**Age**	0.99 (0.98-0.99)	0.99 (0.99-1.00)^1^
**Male gender**	1.59 (1.40-1.82)	1.56 (1.37-1.79)
**Homelessness last 30 days**	1.19 (1.09-1.29)	1.15 (1.05-1.25)
**Lifetime history of psychiatric problems**		
Hospitalization	1.13 (1.02-1.25)	1.11 (1.00-1.23)^1^
Suicide attempt	0.98 (0.89-1.08)	0.97 (0.88-1.07)
Depression	0.94 (0.86-1.04)	0.95 (0.86-1.04)
Anxiety	0.98 (0.90-1.08)	0.99 (0.90-1.09)
Cognitive problems	1.06 (0.98-1.15)	1.05 (0.97-1.14)
Hallucinations	1.02 (0.92-1.14)	1.01 (0.90-1.13)
Difficulty controlling violent behaviour	1.19 (1.09-1.28)	1.21 (1.11-1.31)
**Lifetime history of substance use (>6 months)**		
Amphetamine, heroin and/or injection drug use^2^		
- injecting, no heroin/amphetamine	1.48 (1.17-1.88)	1.54 (1.21-1.95)
- heroin use, no injecting	1.44 (1.12-1.85)	1.44 (1.12-1.86)
- heroin use, injecting	1.88 (1.55-2.27)	1.84 (1.52-2.23)
- amphetamine use, no injecting	1.26 (1.11-1.44)	1.28 (1.13-1.45)
- amphetamine, injecting	1.99 (1.78-2.22)	1.95 (1.74-2.18)
- heroin and amphetamine, no injecting	1.41 (1.05-1.89)	1.43 (1.07-1.93)
- heroin and amphetamine, injecting	2.12 (1.84-2.44)	2.08 (1.81-2.40)
Binge drinking	0.90 (0.83-0.97)	0.89 (0.83-0.97)
Other opioids than heroin/methadone	0.86 (0.77-0.97)	0.85 (0.76-0.95)
Sedatives	0.98 (0.90-1.08)	0.98 (0.90-1.07)
Cocaine	1.01 (0.91-1.13)	1.01 (0.90-1.12)
Cannabis	0.93 (0.85-1.01)	0.93 (0.86-1.01)
Hallucinogenic drugs	0.84 (0.75-0.95)	0.86 (0.76-0.96)
**Number of substance types used last 30 days**	1.05 (1.01-1.09)	1.04 (1.00-1.08)^1^
**Duration of index sentence**	0.93 (0.91-0.94)	0.93 (0.92-0.95)
**Main crime in index verdict**		
Violent crime		0.77 (0.68-0.87)
Property crime		1.19 (1.08-1.32)
Drug crime		0.83 (0.74-0.92)

^1^ Association is statistically significant; confidence interval rounded off to two decimals but separated from 1.

In order to avoid too high correlations between different variables entered as potential predictors, a correlation matrix was run for all variables. The highest correlations were between depression and anxiety (Pearson’s correlation 0.57), between sedative use and number of substance types (0.46), and between property crime and drug crime (0.44), whereas the large majority of correlations were below 0.20. All statistical analyses were carried out in the software SPSS version 17.0.

## Results

Clients were followed for an average of 997 days, 2.7 years (593 days, 1.6 years, for clients returning, and 1,712 days, 4.7 years, for clients not returning). Eighty-nine percent of the included clients were men, and the mean age of clients at the baseline interview was 33.2 years (SD 9.9, median age 32, range 18–73 years). During follow-up, 69 percent (n = 2,862) of clients returned to the criminal justice system. The prevalence figures of each variable included, among clients with and without relapse, respectively, are seen in Table 1. In the Cox regression, amphetamine use, heroin use and injection drug use all predicted recidivism, and the most pronounced risk was seen for clients reporting both amphetamine, heroin and injecting. Also, recidivism was associated with a higher number of substances used prior to incarceration. In addition, the following variables were independently associated with relapse: younger age, male gender, homelessness, previous psychiatric hospitalization, difficulty controlling violent behaviour, and a shorter index sentence, and negatively associated with binge drinking, other opioids, and hallucinogenic drugs. In the second model, when controlling for the type of crime, all significant associations remained, and recidivism was also positively associated with property crime and negatively associated with drug crime and, more strongly, with violent crime (Table 2).

## Discussion

The present study aimed to identify variables, including substance-related and psychiatric problem variables, which predict return to the criminal justice system in prisoners with substance use problems. Several substance-related risk factors were seen, even when controlling for type of crime and the duration of the index sentence. Return to the criminal justice system was predicted by the use of heroin and amphetamine, rather than with other substances, and a more pronounced increase in risk was seen for clients with a history of injecting drug use. Also, recidivism was associated with a higher number of used substances, as well as with a history of psychiatric in-patient treatment, difficulty controlling violent behaviour, male gender, and homelessness.

The association of criminal recidivism with male gender is consistent with previous data [8,27], and poor living conditions previously also have been demonstrated to increase the risk of relapse into crime [8]. Also, psychiatric morbidity, including psychiatric in-patient treatment, previously has been shown to increase the risk of criminal relapse [9,27], although this finding has not been consistent in the literature [8]. Associations with psychiatric in-patient treatment and difficulties controlling violent behaviour were relatively weak in the present study.

The main purpose of the present paper was to study the risk of criminal recidivism related to substance use problems. It is very likely that the types of crimes committed vary depending on the primary drug used by a client, but in the present study, even when controlling for the main types of crimes for which clients were sentenced, associations with some particular features of substance use remained as significant predictors of returning to the criminal justice system. The present study demonstrated independent increases in risk for both of the most commonly injected drugs in the present setting, amphetamine and heroin. In addition, the role of injection drug use was demonstrated, and appears to add some further increase in risk of recidivism, over and above the risk conferred by heroin or amphetamine use alone. The association between injection drug use and criminal recidivism may be mediated by other factors related to both constructs, possibly indicating that a higher degree of deviant behaviour may involve injection drug use and may also be associated with a worse outcome with respect to criminality. However, there also have been data indicating that criminality was actually rather predicted by non-injection use of heroin [28], indicating that the link between injection drug use and criminal behaviour may need further focus in research.

The link between amphetamine use and criminal recidivism has been sparsely addressed in previous literature. There are some data indicating an association in methamphetamine users, although in a different setting and a younger group of methamphetamine users compared to the amphetamine users in the present study [29]. Sweden has seen a long history of a dominating abuse of amphetamine, in contrast to many other countries [30], including high rates of amphetamine use in the criminal justice population, making this the leading substance in the dataset studied here [21]. More remains to be understood about the mechanisms mediating the connection between amphetamine and crime, but the elevated risk of relapsing into crime may have important implications for follow-up and treatment of primary amphetamine users. The development of effective treatment for stimulant addiction historically has been difficult. The last few years have seen emerging scientific support for naltrexone treatment in amphetamine addiction [31], although to date, no medication is formally approved for this type of addiction.

In addition to amphetamine, an elevated risk of relapse was seen also for clients with a history of heroin use, and for clients reporting a higher number of substances used prior to incarceration. The association between heroin use and criminal behaviour is well-known, and the link with recidivism may not be surprising given the high cost of using heroin regularly [3]. Also, treatment of heroin addiction has been shown to decrease the risk of criminal relapse [32].

A significant association was also seen between polysubstance use at baseline and criminal recidivism, with an increase in the risk of recidivism for a higher number of substances used prior to incarceration. While polysubstance use previously has demonstrated a connection to severity of substance use in several other aspects, including mortality, suicide [33,34] and, in the present dataset, higher rates of psychiatric problems [22], the association between polysubstance use and criminal recidivism appears to be a novel finding. Factors mediating this connection are unclear, but may possibly be related to a generally higher severity of substance use complications in subjects who use several substances. Reasons for using multiple substances may differ, and may include both the desire of combining drug effects, and the use of one substance in order to control consequences of the use of another drug [35].

However, the characteristics of individuals with a high degree of polysubstance use are insufficiently understood, and further research may be required in this field. The principal purpose of the present study was to assess the role of substance use variables in the prediction of criminal recidivism when controlling for other factors such as type of crime at baseline. Therefore, type of crime at the index sentence was used mainly as a co-variate to control for in the analysis of potential predictors related to substance use. Likewise, the duration of the index sentence was included in the model as a theoretical proxy variable of severity of the index crime, and displayed an association between recidivism and a shorter time spent in incarceration. The positive association between criminal recidivism and baseline property crime, and the negative associations with baseline violent crime or drug crime, merit further research in order to better understand mechanisms mediating these findings. Relapse rates in clients with different types of criminality may be related to several conditions. For instance, the type of follow-up and treatment referrals offered to clients at prison release may differ according to the type of crime committed before the sentence. There is a paucity of research assessing risk factors of violent recidivism in prisoners, and some of the knowledge in this area comes from research in forensic psychiatric patients. In that setting, a violent index crime has tended to be associated with a lower risk of criminal recidivism [12], but rates of violent re-offending have been shown to be higher in prisoners than in forensic psychiatric patients [13]. In comparison to other Swedish data on relapse rates in violent offending [13], any criminal recidivism was more common in our study, where a majority of clients with a violent index crime returned to the criminal justice system. However, we have little previous data on the risk of relapse in specific types of crime in prisoners with substance use, and there is reason to believe that re-offending may be more common in substance users than in other criminals [13]. In the present study, property crime was associated with a higher risk of returning to the criminal justice system. In previous descriptive data from the Swedish criminal justice system [36], although with no further analysis of risk factors, exceptionally high rates of recidivism were reported in sub-groups of clients. That report pointed out property crime as an index offense very often associated with future relapse; relapse rates in male prisoners with previous convictions even reached above 80 percent. However, subgroups of subjects sentenced for drug crimes also had very high rates of recidivism [36], whereas in the present study, an index drug crime was relatively protective of recidivism compared to the rest of the database. More research may be required in order to improve the understanding of the associations between baseline criminality and relapse. In the previous statistics reported from the whole criminal justice population, regardless of the type of index conviction, rates of recidivism were reported to be 40 percent within three years, only a slightly longer period of time than the average follow-up time in our study. In the present study, rates of recidivism were higher, 69 percent. The present analysis included only prisoners and only substance users, which may have identified a subset of high-risk individuals with respect to relapse into criminal behaviour. Again, the factors mediating these associations may related to characteristics of a specific substance-using prison population, and clearly, there is need for more research about factors mediating the associations between index crime and prospective risk of recidivism.

### Limitations and strengths

The present study has several limitations. First, baseline data are based on self-reported data, which are associated with a risk of recall bias, especially given the time elapsed between prison entry and the interview. Also, while baseline substance use and other characteristics are used as predictors of future criminal recidivism, we did not have access to data describing the situation preceding the relapse into criminal behaviour. Also, from the data available, we were unable to assess the number of future relapses, but rather followed clients from release from prison until the next entry into the criminal justice system or until data were censored. In addition, the present data are based on clients assessed by prison staff as part of a national struggle to improve client-specific and nationwide assessment and interventions with respect to the substance use situation in Swedish criminal justice clients, and therefore, the database studied here is not based on a randomized selection or on any other type of systematic selection, which is a limitation. For reasons related to the overall purpose of the implementation of ASI assessments in this setting, drug users were relatively more common compared to problematic alcohol users, compared to substance users in the whole prison system.

Also, clearly, this kind of registry study can not describe all criminal acts for which a client is never sentenced, and as some offenses may be more likely than others to lead to a new verdict, registry data from the criminal justice system can not fully reflect the true extent and features of criminal behaviour.

On the other hand, the present study is based on a relatively large dataset, and enables the analysis of a large number of potential predictors of criminal relapse. This includes the prospective follow-up design with predictors derived from baseline assessments with a well established interview instrument [15-19], including relatively detailed data on substance use pattern prior to incarceration, making it possible to indicate problem areas that need assessment, intervention and follow-up in criminal justice clients with substance use problems.

## Conclusions

Criminal recidivism was associated with heroin and amphetamine use, injection drug use, and with polysubstance abuse, rather than with alcohol, opioid analgesics and hallucinogenic drugs. The present findings suggest further focus on treatment referrals, evidence-based addiction treatment and structured follow-up of prisoners’ substance use problems. Also, the present findings suggest further research investigating the mechanisms mediating criminal behaviour in clients with different types of substance use patterns.

## Competing interests

No competing interests are reported.

## Authors’ contributions

AH and MB jointly planned the project and the analyses. AH carried out the statistical analyses. AH and MB jointly wrote the manuscript and both approved its final version.

## Pre-publication history

The pre-publication history for this paper can be accessed here:

http://www.biomedcentral.com/1471-244X/12/111/prepub
